# Who Gets the Flu? Individualized Validation of Influenza-like Illness in Urban Spaces

**DOI:** 10.3390/ijerph20105865

**Published:** 2023-05-18

**Authors:** Shiran Zhong, Fenglong Ma, Jing Gao, Ling Bian

**Affiliations:** 1 Department of Geography, University of Western Ontario, London, ON N6A 3K7, Canada; szhong57@uwo.ca; 2 College of Information Sciences and Technology, Pennsylvania State University, University Park, State College, PA 16802, USA; 3 School of Electrical and Computer Engineering, Purdue University, West Lafayette, IN 47907, USA; 4 Department of Geography, University at Buffalo, The State University of New York, Buffalo, NY 14261, USA

**Keywords:** individualized validation, influenza-like illness, transmission-driving factors, urban spaces

## Abstract

Urban dwellers are exposed to communicable diseases, such as influenza, in various urban spaces. Current disease models are able to predict health outcomes at the individual scale but are mostly validated at coarse scales due to the lack of fine-scaled ground truth data. Further, a large number of transmission-driving factors have been considered in these models. Because of the lack of individual-scaled validations, the effectiveness of factors at their intended scale is not substantiated. These gaps significantly undermine the efficacy of the models in assessing the vulnerability of individuals, communities, and urban society. The objectives of this study are twofold. First, we aim to model and, most importantly, validate influenza-like illness (ILI) symptoms at the individual scale based on four sets of transmission-driving factors pertinent to home–work space, service space, ambient environment, and demographics. The effort is supported by an ensemble approach. For the second objective, we investigate the effectiveness of the factor sets through an impact analysis. The validation accuracy reaches 73.2–95.1%. The validation substantiates the effectiveness of factors pertinent to urban spaces and unveils the underlying mechanism that connects urban spaces and population health. With more fine-scaled health data becoming available, the findings of this study may see increasing value in informing policies that improve population health and urban livability.

## 1. Introduction

Urban dwellers are exposed to communicable diseases, such as influenza, in various urban spaces [1,2,3]. Many communicable disease models have been developed to assess health risks in urban settings [4,5,6]. These models involve two processes: prediction and validation. The prediction estimates the health outcomes, and the validation assesses the efficacy of models before they can be used to forecast epidemics or pandemics [7,8]. The two processes can be applied to two scales: population and individual. The scale of prediction refers to that of model output, while the scale of validation conforms to that of model utility [9]. The two processes and two scales render four types of models in terms of the process-scale pairing: (1) population-scaled prediction vs. population-scaled validation, (2) population-scaled prediction vs. individual-scaled validation, (3) individual-scaled prediction vs. population-scaled validation, and (4) individual-scaled prediction vs. individual-scaled validation.

The first type refers to the models built to predict and validate both at a population scale. This type typically includes the family of susceptible–infected–recovered (SIR) models and their various derivatives. The population-scaled focus makes these models most effective in forecasting large-scale health risks, such as pandemics [10,11,12,13]. The second type, population prediction vs. individual validation, refers to the models built at a population scale but validated at an individual scale. This type barely exists, as population-scaled prediction output cannot be directly downscaled to an individual scale for validation.

The third type refers to the models built to predict at an individual scale but validated at a population scale, typically the widely-used agent-based models [14,15,16,17,18,19,20]. Individualized attributes and behaviors and their associated infection probability are used to predict individualized health outcomes. Unmatched to the prediction scale, these models are almost always validated at a coarser scale. The predicted individual health outcomes are aggregated to coarser modeling units (e.g., an entire population), coarser spatial resolutions (e.g., an entire study area), or coarser temporal resolutions (e.g., an entire epidemic) for validation. The unmatched scales remain unresolved due primarily to the scarcity of ground truth data that can support the desired validation. This issue significantly undermines the efficacy of these individual prediction models in assessing the vulnerability of individuals, communities, and urban society at large. In this sense, the fourth type, i.e., models that are both built and validated at an individual scale, is much needed, although extremely rare.

The transmission of communicable disease is inherently a spatial process, arising in three typical urban spaces: the home space, work space (including schools), and service space (e.g., supermarkets and healthcare facilities). Various individualized, spatially explicit transmission-driving factors have been considered in individual models [14,18,21,22,23]. The effectiveness of the factors remains un-substantiated due to the lack of individual-scaled validation. Knowledge gained from individual models and the effectiveness of the factors is paramount to identifying vulnerable individuals and communities and where and when they can be affected. Such insights may be invaluable to support spatially and temporally informed intervention to contain localized outbreaks at the early stage of potential epidemics or pandemics.

The objectives of this study are twofold. First, we aim to model and, most importantly, validate influenza-like illness (ILI) symptoms at the individual scale based on a set of transmission-driving factors pertinent to urban spaces. The effort is supported by an ensemble approach. Second, we investigate the effectiveness of the factors through an impact analysis. The research leverages data from a large number of individuals during an influenza season in a metropolitan area in the northeastern U.S.

This study contributes to urban health studies from two perspectives. First, the results provide evidence that health outcomes can be validated at the individual scale. This paves the way for efforts to validate a large number of existing and future models. Second, the individualized validation substantiates the effectiveness of transmission-driving factors pertinent to urban spaces. The findings reveal the connections between urban spaces and health risks and help devise strategies that help vulnerable individuals and communities in urban society.

## 2. Literature Review

### 2.1. Transmission-Driving Factors

Influenza is transmitted primarily through three mechanisms, according to the CDC and numerous studies [24,25,26]. The first mechanism is direct contact between infectious and susceptible individuals. The second and third types are droplet transmission and airborne transmission, respectively, where small particles are dispersed into the air by infectious individuals and inhaled by susceptible individuals. For the third mechanism, the dispersed particles are extremely small and can remain suspended in the air for extended periods of time. These transmissions are most relevant in indoor environments where individualized face-to-face interactions easily cause infections [27].

The influenza transmission between individuals may differ in the three typical urban spaces. At home and workplaces, the interactions are highly frequent, long-lasting, and consistent, inducing local infections between family members and between co-workers. Regular commuting between home and workplaces connects local infections to a broader home–work space. At service places, individuals engage in activities outside homes and workplaces, such as shopping and seeking healthcare. Unlike in the home–work space, the interactions in the service space are highly random, dynamic, and diverse. The severity of the risks, however, remains unknown, although empirical evidence begins to indicate the linkage between service place visits and health risks [1,6,13,28,29,30,31].

The transmission-driving factors considered in individual models include individualized and spatially explicit attributes and behaviors [32,33,34]. These attributes typically include individualized demographic, exposure environment, and associated infection probabilities, while the behaviors include mobility, interaction, and protective actions against infection. The probabilities associated with the attributes and behaviors are believed to vary with location and time [35,36,37]. The effect of the factors on individual health outcomes, however, has not been substantiated. Additionally, transmission-driving factors at the community level represent a backdrop exposure environment that facilitates individualized risks. This is especially the case in highly populated metropolitan areas, although the factors are not commonly considered in individual models [38]. Studies have considered the block group as an appropriate proxy to characterize community-level factors [39,40,41,42,43].

Ambient environments, such as temperature and air quality, are proven effective in predicting influenza epidemics [44,45,46,47,48,49]. These factors are not individually or spatially resolved and are found effective in predictions using coarse modeling units (e.g., population) or at a coarse spatial resolution (e.g., county), yet are under-exploited in individual models. Being temporally resolved, the ambient environmental factors could be invaluable in representing the undercurrent that drives individualized factors through an influenza season. Commonly used temperature data in influenza studies include daily, weekly, or monthly averages of hourly recorded temperature. The commonly used air quality data is a composite index that synthesizes PM25, O_3_, NO_2_, and CO measures [50,51,52].

### 2.2. Data Issues and Modeling Approaches

In recent years, individualized health outcome data have gradually become available [6,23,53,54,55], including those for ILI symptoms. With this development, challenges arise due to the nature of such data, for example, the imbalance between the majority data records and the minority data records. In the case of ILI data, the records showing the absence of symptoms are much more than that showing the presence of symptoms. The imbalance may bias the prediction process and cause unstable and unreliable results [56,57].

The most commonly adopted approach to dealing with data imbalance is naively oversampling the minority records [58,59]. The simplistic approach inevitably brings limitations, such as model overfitting during the training process and a loss of flexibility during the testing process, especially when handling complex datasets. An approach to minimizing the effect of imbalanced data is desired when validating the individualized health outcome data.

Method-wise, machine learning approaches have been employed more recently in individual models to predict fine-scaled health outcomes [60,61,62,63,64,65]. These approaches include logistic regression, support vector machine (SVM), decision tree, random forest, artificial neural network (ANN), and convolutional neural network (CNN). Among them, both logistic regression and SVM are designed for dichotomous classifications, although through different mechanisms. Logistic regression establishes the statistical relationship between dichotomous states (e.g., presence and absence) and explanatory factors. SVM predicts the dichotomous state by solving a hyperplane in high-dimensional factor space [66,67].

A decision tree is designed for multi-state prediction through a hierarchical decision tree structure. The prediction process assesses the relationship between the state and a suite of factors while randomizing the factors through the hierarchy. Random forest, on the other hand, employs an ensemble of decision trees and considers the majority votes of the tree assessment as the final prediction. The ensemble of trees allows the randomization of factors between trees, and this design effectively exploits information in the data [68,69]. ANN and CNN are designed for classification by capturing the intricate nonlinear relationship between states and factors. ANN utilizes layers of neurons to assign weights to factors to approximate the states in order to capture the relationship. CNN uses convolutional kernels to synthesize weights so that they are most representative of high-dimensional factors to capture the relationship [70,71]. However powerful, none of these approaches were originally designed to handle imbalanced data, and the modeling process may be biased toward the majority absence records if employed in a naive manner.

## 3. Materials

### 3.1. Source Data

The data used for this study are primarily derived from a smartphone-based survey. The remaining data are obtained from a number of meteorological and air monitoring stations in the study area. The survey was conducted on a weekly basis during the 2016–2017 influenza season in a metropolitan area in the northeastern U.S. It was approved by the Institutional Review Board (IRB) at the University at Buffalo. The survey collects two types of data: individualized health outcome data and individualized attribute and behavior data relevant to disease transmission. More than 2000 individuals participated in the survey from late October to late May (of the following year) based on the influenza season defined by the CDC. Individuals whose residential locations fall within the metropolitan area are included in the study. From these individuals, only those who participated continuously for two months or more are further selected. Next, individuals who reported continuous ILI symptoms for a period within the CDC guidelines are considered valid [24]. The filtering process resulted in a total of 1485 individuals for the subsequent analysis. The large-size and long-term data collection effectively supports the need for the intended prediction and validation.

The health outcome data are the presence and absence of ILI symptoms in individuals. The data serve as the ground truth to support the intended validation. The ILI symptoms include fever, cough, sore throat, runny nose, body aches, headaches, or fatigue [24,72]. For influenza transmission, the infectious period typically lasts up to four weeks (including a latent period and a subsequent symptomatic period). A susceptible individual could be infected anytime during the period if in contact with infectious sources. Once infected, the individual may show symptoms for typically two weeks [24,72,73]. The health outcome data collected on a weekly basis is able to capture both possible infecting sources and the presence of symptoms.

The survey also provides a list of health-related, individualized attribute and behavior data to support the representation of transmission-driving factors. These include individualized demographic, exposure, mobility, interaction, and protective action information. The survey data involved in this study are listed in Appendix A.

Data obtained from the meteorological stations and air monitoring stations are used to support the representation of ambient environmental factors. The temperature data are retrieved from two meteorological stations in the study area administrated by the National Weather Service [74]). The composite air quality index that synthesizes multiple air quality measures (PM25, O_3_, NO_2_, and CO) is obtained from three air monitoring stations powered by the New York State Department of Environmental Conservation [75].

### 3.2. Transmission-Driving Factors

From the source data, information is extracted to represent four sets of transmission-driving factors related to (1) the home–work space, (2) the service space, (3) the ambient environment, and (4) demographics.

The first set of factors characterizes the health risks pertinent to the home–work space at the individual and community levels. The individual-level factors include whether individuals’ close contacts showed ILI symptoms during a week, where the close contacts include family members, co-workers, and other face-to-face contacts in the home–work space. The set also includes the protective actions taken by the individuals. These include getting a flu shot, staying home from work (school), seeing a doctor, and taking over-the-counter medicine. Both contact exposure and protective actions directly affect health risks. These factors are encoded as Boolean features.

The risk at the community level includes the total number of individuals who commute to work or visit service places in an individual’s home block group, the total number of individuals who reside or visit service places in an individual’s workplace block group, the total number of ILI cases in an individual’s home block group, and the total number of ILI cases in an individual’s workplace block group. These factors provide a local context of exposure as communities of active interactions may enhance individual health risks. According to the literature, the block group is appropriate to characterize the community-level risks (See Section 2). These factors are encoded as numeric features.

The second set of factors considers risks related to the service space from both an individual-centric and a place-centric perspective. The individual-centric factor is the total number of visits to service places made by individuals, as frequent visits to a random, dynamic, and diverse interaction environment may expose individuals to high risks. The place-centric factors include the “popularity” of visited service place types and the presence of symptomatic cases at a type (see Appendix B for a full list of place types and their popularity). Populated place types may present a higher health risk than less-populated ones due to the high-volume interaction environments. The number of symptomatic cases at a service place type represents direct health threats. A total of 89,260 service place visits are extracted from the survey, and they belong to 12,985 service places in 32 types. Typical place types include supermarkets, healthcare facilities, department stores, gyms, churches, cafes, and others (Appendix B). The factors related to the service space are encoded as numeric features.

The third set of factors refers to the ambient environment: temperature and air quality. The factors include the weekly average of the hourly recorded temperature and the weekly average of the composite air quality index. Because all stations are located within the metropolitan area, the measured values are extremely similar between stations for both types. The average value across stations for the respective type is deemed appropriate and used for this study. The ambient environmental factors are encoded as numeric features.

The last set refers to commonly used demographic factors, including age group, gender, and household size of individuals. The demographic factors are denoted as categorical features, except household size as a numeric feature.

### 3.3. Ground Truth for Validation

The presence or absence of ILI symptoms in individuals is used as the ground truth for validation. For all 1485 individuals each week, a total of 25,221 (individual-week) records are derived from the source data. Of these, 2756 are presence records, and the remaining 22,465 are absence records. The four sets of transmission-driving factors in the prior four weeks are used in the model to predict the presence or absence of ILI symptoms in individuals in the following two weeks. The prediction is validated by comparing the predicted presence or absence of ILI symptoms in individuals with the observed ground truth records. The effectiveness of the transmission-driving factors is assessed in the subsequent impact analysis.

## 4. Methods

### 4.1. Ensemble Approach

An ensemble approach is devised to support the first objective of the study, i.e., predicting and validating ILI symptoms at the individual scale while addressing the data imbalance issue. The approach integrates three method components: the ensemble design of random forest as the frame, the dichotomous classifiers of SVM as the building block, and a “train-small-evaluate-big” strategy for training. The integration of the components is based on their design strengths (See Section 2.2 The train-small-evaluate-big strategy is detailed in the following text) and the context of this study.

**Figure 1:**
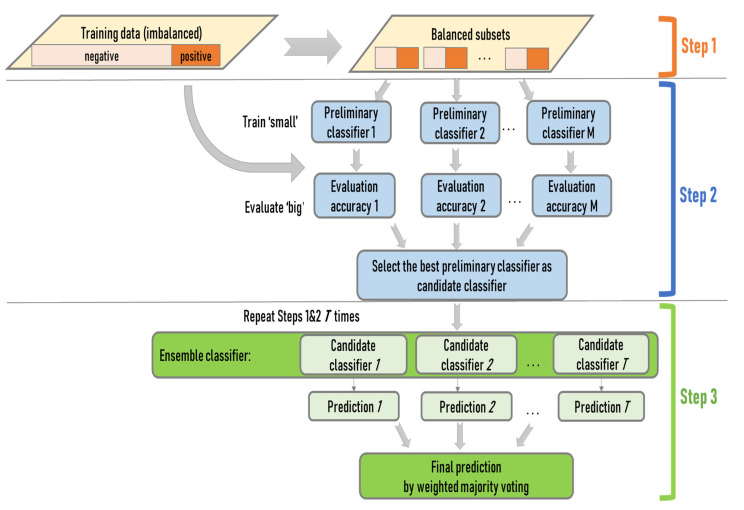
The schematic workflow of the ensemble approach.

The ensemble approach consists of a training and a testing process. The training process captures the relationship between the presence (absence) of ILI symptoms and transmission-driving factors, while the testing process predicts the presence (absence) of ILI symptoms based on the captured relationship. The train-small-evaluate-big strategy is implemented in the training process. The training data (including both the factor data and the corresponding ILI symptoms data) are iteratively partitioned into multiple training sets (Figure 1). For each set, the presence records are bootstrapped with replacement to generate multiple subsets. Meanwhile, the absence records are randomly divided into multiple subsets, with the number of subsets and the size of each subset the same as those of the present data. Then, a presence subset is paired with an absence subset to generate a small balanced subset. The pairing generates multiple sets of small balanced subsets for each training set.

To build the ensemble of SVM classifiers, a large pool of preliminary SVM classifiers is trained from the small balanced datasets, with one classifier from each subset (train small). The trained classifiers are applied to the entire training data to evaluate their generalizability (evaluate big). The classifier with great generalizability is selected from each training set, and the collection of best-performing classifiers from all training sets forms the ensemble. Lastly, the ensemble of classifiers is applied to the testing data to predict the presence or absence of ILI symptoms through a weighted majority voting. The classifiers in the ensemble that perform better during the training carry greater weights in the voting.

The iterative randomization at multiple stages of the ensemble approach (training set partition, sub-dataset balance, SVM classifier selection, and ensemble composition) is intended for full consideration of both majority and minority information in the data. The design avoids arbitrarily excluding information, as in naive oversampling. On the other hand, the selection of the best classifiers from a large number of small balanced sub-datasets engages the most representative information in the data. The weighted majority voting may prevent undue effects of outliers possibly brought in by the full consideration of all information.

### 4.2. Model Setting

A number of parameters are involved in the training process. Among them, the training-versus-testing division, the replacement rate for the presence subsets, and the number of training sets are most critical. These parameter values are determined according to the literature or selected through experiments. First, the training-versus-testing division is set by ratios of 80 versus 20 and 70 versus 30, according to the literature [76,77]. The 80 versus 20 ratio is the most common choice, while 75 versus 25 is occasionally used. The 70 versus 30 division ratio used in this study is more rigorous and rare. Second, the replacement rate for the presence subsets is set at 0.75, based on an experiment of a series of alternative rates to achieve a stable result. Third, the number of training sets is set as 45, where the training process converges after experimenting with the number from 1 to 100, with an increment of 1.

The performance of the ensemble approach is compared with established machine learning approaches. Six such models are employed as the benchmark, including logistic regression, standalone SVM, decision tree, classic random forest, ANN, and CNN. Logistic regression uses the limited-memory Broyden–Fletcher–Goldfarb–Shanno (L-BFGS) for optimization [78]. For standalone SVM, the kernel function adopts the radial basis function, the same as that used in the ensemble approach. For the decision tree, the maximum depth of the tree is set as 10, according to the literature [76]. For the classic random forest, the maximum tree depth is also 10. For ANN, the number of hidden layers is set to three. For CNN, the initialization of convolution kernels follows a random Gaussian distribution. The values of the hyper-parameters, including weight decay and learning rate, are selected following the comprehensive guidelines presented by [77] to facilitate training convergence. The six benchmark models use the same training-versus-testing division ratio as the ensemble approach.

### 4.3. Model Validation

To validate the individual model, the predicted presence and absence of individual ILI symptoms are compared with the observed ground truth records. Both the overall accuracy and F1 scores are used to convey validation results. The overall accuracy provides a synoptic sense of performance; it refers to the number of correctly predicted records out of the total number of records in the testing data. The F1 score offers a more rigorous measure because it combines both precision and recall, where the precision accounts for how many records are correctly predicted among all the predictions, while the recall accounts for how many records are correctly predicted out of all the observed records [79,80]. The F1 ranges from 0 to 1, with 1 meaning perfect precision and recall and 0 meaning a failure in prediction. Validation is applied to the ensemble approach and the six benchmark models.

### 4.4. Factor Impact Analysis

To support the second objective of evaluating the effectiveness of transmission-driving factors, the four sets of factors are removed from the model in different combinations. The changes in prediction accuracy reveal to what extent the removed factors contribute to the prediction, where a greater decrease in prediction accuracy indicates a higher contribution. Two removal strategies are employed: a single-set strategy and a multi-set strategy. The single-set strategy removes one set of factors at a time while leaving the other three sets intact. The intention is to evaluate the separate contribution of each factor set. The multi-set strategy removes two types of combinations. One type is all possible combinations of two-factor sets out of the total of four sets, yielding six combinations, and the other is all possible combinations of three-factor sets, yielding four combinations. The intention is to evaluate the combined contribution of factor sets. Pearson correlation is conducted among all factors, and results indicate minimal collinearity between them. This ensures an unbiased assessment of factor contribution.

## 5. Results

### 5.1. Results of Individual ILI Validation

The overall accuracy of the ensemble approach reaches a level of 84.1% (Table 1). This accuracy is noticeably higher than those of the five benchmark models, which are consistently lower, close to, or slightly above 80% (note that the CNN benchmark model does not converge and is not able to produce prediction results).

The F1 scores, as a more comprehensive accuracy measure, assess accuracy for both the majority absence and minority presence prediction. For the majority absence prediction, the F1 score by the ensemble approach reaches 95.1%, and similarly high F1 scores are also observed by the five benchmark models. As the absence records are dominant in the raw data, it is not a surprise that the F1 scores remain high across all models. For the minority presence prediction, the F1 score by the ensemble approach reaches 73.2%. This level of accuracy is satisfactorily high, given the considerable imbalance in the raw data (presence records versus absence records ≈ 1:9). The five benchmark models achieve noticeably lower accuracies than the ensemble approach, ranging from 55.0–68.4%. The difference in performance proves the strength of the ensemble approach when handling extremely imbalanced data.

Further, the ensemble approach performs relatively stable across the two training-vs-testing division ratios (Table 2), demonstrating the robustness of the approach. Although the more restricted 70 versus 30 division uses less training data, it can still effectively capture the complex relationship between the presence (absence) of ILI symptoms and transmission-driving factors, suggesting that overfitting or underfitting is minimal. The mispredicted cases show a mixture of over- and under-prediction. No specific factor sets stand out that obviously contribute to the mispredictions.

### 5.2. Results of Factor Impact Analysis

**Figure 2:**
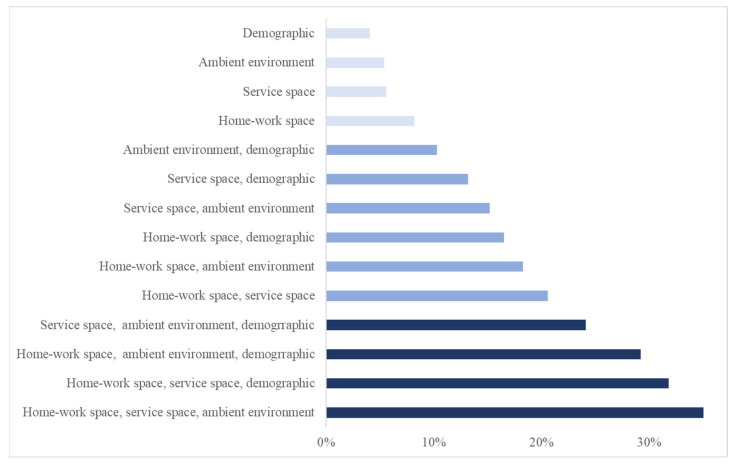
The contribution of transmission-driving factor sets include separate contributions (the top four bars) and combined contributions (others).

For the single-set removal strategy (the four light blue bars at the top of Figure 2), the decrease in F1 scores in each set indicates their separate contributions to the prediction, up to 10% (Table 3). For the six combinations of removing two-factor sets (the six blue bars in the middle of Figure 2), their contributions range from 10~20%. For the four combinations of three-set removal (the four dark blue bars at the bottom of Figure 2), their contributions are in the range of 20~40%.

## 6. Discussion

### 6.1. Accuracy Level of Individual-Scaled Validation

As an early attempt to validate health outcomes at the individual scale, the level of accuracy achieved (84.1% overall, 95.1% for the majority absence, and 73.2% for the minority presence) is satisfactory. Individual-scaled validation is subject to more demanding accuracy assessment due to its lower tolerance to prediction errors than coarser-scaled validation. Every single erroneous prediction case counts either towards over-prediction or under-prediction, and there is no room to offset each other as may happen in coarser-scaled modeling. Further, heterogeneity and randomness associated with individualized attributes and behaviors are more prominent in individual models, and this characteristic subjects individual-scaled validation to lower accuracy than coarse-scaled models. The individual-scaled accuracies attained in this study reach the accuracy level of a large number of models that are validated at coarse scales. In this sense, our results are more than satisfactory given the aforementioned challenges faced in individual-scaled validation [13,81].

Individual-scaled validation is rare, with the exception of Barlacchi et al. [54], where the ILI symptoms are both predicted and validated at the individual scale. As an early attempt, the validation is over a small survey size (29 individuals) and a short period (14 days). Our validation is drawn from a large survey size (1485 individuals) and a long period (an influenza season of more than seven months). This study provides preliminary evidence that the health outcomes can be validated at the individual scale. This undertaking is significant because it paves the way for efforts to validate a large number of existing and future individual models.

### 6.2. Separate and Compound Impact of Factors

Among the separate contributions of the four factor sets (1–10%), the home–work space set contributes the most, 8.2% (Table 3). This reflects the effect of intensive interactions in this indoor space on health risks. A few symptomatic individuals may infect many family members and co-workers, while regular commuting between home and workplaces can rapidly spread infections to various communities. The contribution of the service space factor set, 5.6%, is second to the home–work space. The high-volume face-to-face interactions among a large number of individuals in this indoor space may easily cause infections. Recently, there has been an increased interest in the role of service spaces in disease outbreaks. This study provides valuable evidence regarding health risks unique to the service space that has rarely been identified.

The contribution of the ambient environment factor set, 4.7%, reflects their underlying effect on the prevalence of ILI symptoms at different times of an influenza season. The health risk in the home–work space and service space may be considerably exacerbated by cold temperatures and poor air quality. Cold weathers increase indoor activities and intensify health risks, as influenza is mostly transmitted in indoor environments. Additionally, people are prone to lowered immunity and more severe symptoms in cold weather or when air quality is poor [45,46,82].

As expected, the demographic factor set also contributes to ILI transmission, 4.0%, and the contribution varies across the demographic factors. There are no statistical gender differences with respect to the absence and presence of ILI symptoms (*p* = 0.14). This is consistent with the existing literature that gender has not been found to be predictive of influenza [83]. The young and elderly populations have been perceived as vulnerable groups to infection, but the difference in health outcomes by age group is found insignificant in this study. Household size is significant in differentiating the presence and absence of ILI symptoms (*p* = 0.03). Large households may have multiple children who possibly bring the infection from school to home. By nature, demographic factors are individually and spatially resolved but temporally invariant. Their contribution may not be explicit in dynamic predictions [38,84], and these factors could be more effective in temporally synoptic studies.

Among factor sets, specifically, the contribution of a two-set combination, home–work space and service space (20.5%), is 6.7% higher than the sum of their separate contributions (13.8% = 8.2% + 5.6%). This observation may imply reinforced health risks involved in the two spaces. Although the effect of the home–work space is more dominant, the “supplement” contribution of the service space is noticeable. While in the home–work space, commuting spreads the infection to rather fixed pairs of locations regularly, service places serving as hubs can connect a large number of homes and workplaces dynamically, causing the transmission to a broad range of locations throughout an urban area. These reinforced health risks can be vital to the population’s health in complex urban environments. For all other two-factor set combinations, compound effects are also observed, varying to different degrees.

The contributions of the three-set factors (20~40%) are much greater than the simple arithmetic sum of their separate contributions (14.3~18.5%) and imply a significant compound effect. Of particular note is the combination that involves the two spaces and the ambient environment. The contribution (40.5%) is so substantial that it is more than twice the sum of separate contributions of the three sets (18.5% = 4.7% + 5.6% + 8.2%). The compound effects of the home–work space and service space are doubled by the ambient environment that shapes the dynamics of an epidemic.

As an early undertaking to substantiate transmission-driving factors in individual models, the results of this study provide empirical evidence of their effectiveness, while the effect has been long assumed. The individually, spatially, and temporally resolved factors, as well as their combination, unveil the mechanism that connects urban spaces and population health when urban dwellers interact with complex urban environments. The results of this study bring the theoretical implication of the Modifiable Area Unit Problem [85,86], which is a fundamental principle in GIScience and spatial–temporal analysis. The individual-scaled validation is not simply the validation at “another scale” but may rather involve different processes, driving factors, and results. The investigation of individual-scaled validation presents theoretical and practical questions that warrant serious attention, although an in-depth discussion of these questions falls outside the scope of this study.

## 7. Conclusions

This study predicts and validates the presence (absence) of influenza-like illness (ILI) symptoms at the individual scale based on four sets of transmission-driving factors using an ensemble approach. The validation takes advantage of data on both individualized health outcomes and influenza transmission-driving factors. Because fine-scaled validation has rarely been achieved, results of the fine-scaled prediction vs. fine-scaled validation obtained in this study lay a solid groundwork to support efforts to validate a large number of existing and future individual models. The individualized validation substantiates the effectiveness of four sets of transmission-driving factors arising in typical urban spaces. Findings lay the foundation for efforts to future decipher the complicated nature of the disease dynamics in urban environments.

There are limitations to this study. The ensemble approach is effective for handling imbalanced individual-scaled data and is potentially applicable to other disciplines, such as ecology and criminology, that face data imbalance issues. Advanced approaches, such as deep learning, could be more powerful in dealing with the issue, but they often require restricted conditions to unleash their power. For instance, in this study, the deep learning approach, CNN, does not converge as it demands an extremely large volume of data to reach its full potential. With the increasing availability of individualized health outcome data, the advanced approaches may reach their intended capacity.

Smartphone-based surveys routinely face trade-offs between three considerations: frequency of data collection, size of the survey, and length of the survey period due primarily to battery consumption issues. Presently few smartphone-based surveys can maintain the three considerations simultaneously, and one of the three considerations is usually compromised [87,88]. We choose the weekly data collection and compromise the potential for daily-scaled validation, although the latter can inform real-time intervention strategies. The rapid advances in smartphone technology may eventually resolve this issue.

As an early attempt at individual-scaled validation, the results of this study warrant further exploration in several research areas, such as the design and utility of smartphone-based surveys to collect fine-scaled health outcome data. Similarly, the selection of transmission-driving factors has ample room for further explorations well beyond individualized factors. Collectively, knowledge gained is critical for developing timely interventions, especially at an early stage of localized disease outbreaks. With fine-scaled health data increasingly becoming available, the findings of this study may see increasing value in informing policies that improve population health and urban livability.

## Figures and Tables

**Table 1:** Validation of ILI symptom prediction.

Validation Accuracy	Ensemble Approach	Logistic Regression	Stand-Alone SVM	Decision Tree	Classic Random Forest	ANN	CNN
Overall accuracy	**84.1%**	80.5%	81.1%	78.5%	83.3%	82.7%	Non- converged
F1 for majority absence	**95.1%**	92.5%	94.2%	91.0%	95.0%	95.2	Non- converged
F1 for minority presence	**73.2%**	55.0%	63.3%	60.5%	65.2%	68.4%	Non- converged

**Table 2:** Results of ILI symptom prediction with different training ratios.

Validation Accuracy	Training vs. Testing Ratio	
	80 vs. 20	70 vs. 30
Overall accuracy	**84.1%**	81.5%
F1 for majority absence	**95.1%**	94.3%
F1 for minority presence	**73.2%**	69.2%

**Table 3:** Separate and compound contributions of factor sets.

Factors	Compound Contribution	Sum of Separate Contributions	Extra Contribution
Home–work space	8.2%		
Service space	5.6%		
Ambient environment	4.7%		
Demographics	4.0%		
Home–work space, service space	20.5%	13.8%	6.7%
Home–work space, ambient environment	19.2%	12.9%	6.3%
Home–work space, demographics	16.2%	10.3%	5.9%
Service space, ambient environment	15.5%	12.2%	3.3%
Service space, demographics	12.5%	9.6%	2.9%
Ambient environment, demographics	11.0%	8.7%	2.3%
Home–work space, service space, ambient environment	40.5%	18.5%	22.0%
Home–work space, service space, demographics	30.5%	17.8%	12.7%
Home–work space, ambient environment, demographics	30.1%	16.9%	13.2%
Service space, ambient environment, demographics	26.5%	14.3%	12.2%

## Data Availability

The survey involved in this study includes human research participants. The research has obtained formal approval from the Institutional Review Board (IRB) of University at Buffalo and was conducted in compliance with all IRB decisions, conditions, and requirements. Due to the nature of this research, participants of this study did not agree for their data to be shared publicly, so supporting data is not available.

## Appendix A

The recruitment of the survey followed a stratified randomized design based on a number of demographic considerations according to the census data of the study area. Participants were involved in an online registration and a weekly smartphone survey. The registration collected basic demographic data of participants. At the weekly survey, participants answered a list of questions shown below via a smartphone app installed on their devices. The app was designed for both Android and iPhone.

**Table A1:** List of questions in the smartphone survey.

Phase	Category	Questions
Online registration	Demographic	Age group
		Gender
		Household size
	Address	Residence
		Workplace
Weekly survey	Health Outcomes	Flu-like symptoms during the last week
	Exposure	ILI symptoms of family members
		ILI symptoms of co-workers
		ILI symptoms of other face-to-face contacts
	Mobility and Interaction	Service places visited during last week
	Protective Action	Getting a flu shot last week
		Staying home from work (school)
		Seeing a doctor
		Taking over-the-counter medicine

## Appendix B

**Table A2:** Total Visits to Service Place Types.

Place Type	Total Visit	Popularity *
Supermarket	15,589	0.170
Healthcare	14,520	0.159
Department stores	11,424	0.125
Gym	6700	0.073
Church	6480	0.071
Cafe	4270	0.047
Financial service	3124	0.034
Restaurant	2644	0.029
Bar	2404	0.026
Park	2338	0.026
Furniture store	2061	0.023
Casino	2058	0.022
Home goods store	2052	0.022
Car repair	2015	0.022
Library	1736	0.019
Bakery	1474	0.016
Liquor store	1280	0.014
Stadium	1173	0.013
Dentist	1128	0.012
Bowling alley	913	0.010
Beauty salon	855	0.009
Airport	762	0.008
Veterinary care	693	0.008
Movie theater	651	0.007
Gas station	621	0.007
Bookstore	544	0.006
Car wash	530	0.006
Lodging	464	0.005
Meal takeaway	456	0.005
Jewelry store	280	0.003
Museum	154	0.002
Spa	76	0.001

* The popularity is calculated as a ratio of the total person-day visits received by a place type over the grand total of person-day visits of all types.
